# Pump-Free Microfluidics for Cell Concentration Analysis on Smartphones in Clinical Settings (SmartFlow): Design, Development, and Evaluation

**DOI:** 10.2196/62770

**Published:** 2024-12-23

**Authors:** Sixuan Wu, Kefan Song, Jason Cobb, Alexander T Adams

**Affiliations:** 1 School of Interactive Computing Georgia Institute of Technology Atlanta, GA United States; 2 Wallace H Coulter Department of Biomedical Engineering Georgia Institute of Technology Atlanta, GA United States; 3 Renal Medicine School of Medicine Emory University Atlanta, GA United States

**Keywords:** mobile health, mHealth, ubiquitous health, smartphone, chip, microscope, microfluidics, cells counting, body fluid analysis, blood test, urinalysis, computer vision, machine learning, fluid, cell, cellular, concentration

## Abstract

**Background:**

Cell concentration in body fluid is an important factor for clinical diagnosis. The traditional method involves clinicians manually counting cells under microscopes, which is labor-intensive. Automated cell concentration estimation can be achieved using flow cytometers; however, their high cost limits accessibility. Microfluidic systems, although cheaper than flow cytometers, still require high-speed cameras and syringe pumps to drive the flow and ensure video quality. In this paper, we present SmartFlow, a low-cost solution for cell concentration estimation using smartphone-based computer vision on 3D-printed, pump-free microfluidic platforms.

**Objective:**

The objective was to design and fabricate microfluidic chips, coupled with clinical utilities, for cell counting and concentration analysis. We answered the following research questions (RQs): RQ1, Can gravity drive the flow within the microfluidic chips, eliminating the need for external pumps? RQ2, How does the microfluidic chip design impact video quality for cell analysis? RQ3, Can smartphone-captured videos be used to estimate cell count and concentration in microfluidic chips?

**Methods:**

To answer the 3 RQs, 2 experiments were conducted. In the cell flow velocity experiment, diluted sheep blood flowed through the microfluidic chips with and without a bottleneck design to answer RQ1 and RQ2, respectively. In the cell concentration analysis experiment, sheep blood diluted into 13 concentrations flowed through the microfluidic chips while videos were recorded by smartphones for the concentration measurement.

**Results:**

In the cell flow velocity experiment, we designed and fabricated 2 versions of microfluidic chips. The ANOVA test (Straight: *F*_6, 99_=6144.45, *P*<.001; Bottleneck: *F*_6, 99_=3475.78, *P*<.001) showed the height difference had a significant impact on the cell velocity, which implied gravity could drive the flow. The video sharpness analysis demonstrated that video quality followed an exponential decay with increasing height differences (video quality=100*e^–k×Height^*) and a bottleneck design could effectively preserve video quality (Straight: R^2^=0.95, k=4.33; Bottleneck: R^2^=0.91, k=0.59). Samples from the 13 cell concentrations were used for cell counting and cell concentration estimation analysis. The accuracy of cell counting (n=35, 60-second samples, R^2^=0.96, mean absolute error=1.10, mean squared error=2.24, root mean squared error=1.50) and cell concentration regression (n=39, 150-second samples, R^2^=0.99, mean absolute error=0.24, mean squared error=0.11, root mean squared error=0.33 on a logarithmic scale, mean average percentage error=0.25) were evaluated using 5-fold cross-validation by comparing the algorithmic estimation to ground truth.

**Conclusions:**

In conclusion, we demonstrated the importance of the flow velocity in a microfluidic system, and we proposed SmartFlow, a low-cost system for computer vision–based cellular analysis. The proposed system could count the cells and estimate cell concentrations in the samples.

## Introduction

### Overview

Cell counting and concentration analysis are common practices to help us understand a variety of health conditions. This includes counting cells where they are supposed to be, in our blood, where a low red blood cell count can suggest conditions such as anemia [1], which can lead to fatigue and other serious complications if untreated. Similarly, elevated white blood cell counts can be a marker of infections or hematological diseases like acute leukemia [2]. Cell counting is also used to look for cells where they should not be, such as our urine. Elevated red blood cell concentration in urine samples serves as an indicator of hematuria [3], which is an important indicator of kidney disease, such as acute kidney injury and chronic glomerular diseases [4]. Accurate and accessible measurement of cell concentrations is therefore critical in both clinical diagnostics and ongoing patient management.

Estimating cell concentrations in samples can be achieved with both manual and automated methods [5]. The slide method requires clinicians to separate the sample into components with a centrifuge, prepare sample slides, conduct observations under a microscope, and manually count the cells [6]. This method demands a high level of expertise from clinicians, is subject to human error, and can be labor-intensive [7]. An advancement in this method is the imaging cytometer (eg, SpectraMax MiniMax 300, Molecular Devices LLC) [8]. This incorporates analysis software and image processing to remove the need for staining in most use cases. These devices cost approximately US $50,000 and still have many of the same issues as manual methods. In addition, automated methods for estimating cell concentration can involve the use of flow cytometers [9]. In this technique, cells flow through a narrow beam of light, and the scattering and fluorescence signals produced by the cells are measured to obtain information about their properties. However, flow cytometers are expensive (between US $100,000 and US $500,000) and not as readily accessible as microscopes in laboratory environments.

As microfluidic technologies become increasingly prevalent, researchers are exploring the feasibility of automating cell concentration analysis using microscopes coupled with microfluidic platforms. Although these automated microfluidic chips are more accessible than traditional flow cytometers, they still require costly high-speed cameras and syringe pumps to control flow rates and ensure video quality. These requirements make microfluidic systems less accessible and could hinder the widespread adoption of computer vision–powered microfluidic medical devices [10,11].

To enhance the accessibility and affordability of automated cell concentration estimation, we introduced SmartFlow. Different from other microfluidic platforms, there is no syringe pump nor high-speed camera setup in the SmartFlow design. Syringe pumps are commonly used to drive the flow in microfluidic platforms. On the other hand, SmartFlow leverages gravity to drive the flow. Moreover, instead of using high-speed cameras, smartphones are used for video recordings in SmartFlow. To preserve the video quality, we introduced a bottleneck design for the microfluidic channel by slowing down the flow velocity. Therefore, a high-speed camera is not necessary in the SmartFlow setup. The cost of the 3D-printed microfluidic chip was around US $15, while smartphones and microscopes are common and prevalent equipment in clinical settings. Therefore, SmartFlow is much cheaper and more accessible than commercial flow cytometers and other microfluidic systems.

SmartFlow can measure cell concentrations within a mean absolute percentage error of 25% compared with the gold standard. Our evaluation of SmartFlow covered a broad spectrum of concentrations, ranging from those exceeding upper bounds in blood to levels as low as those found in healthy urine samples. In this paper, we answered the following research questions (RQs): RQ1, Can gravity drive the flow within the microfluidic chips, eliminating the need for external pumps or pressure systems? RQ2, How does the microfluidic chip design impact video quality for cell analysis? RQ3, Can smartphone-captured videos be used to estimate cell count and concentration in microfluidic chips?

We designed and conducted a cell flow velocity experiment and cell concentration analysis experiment to validate 3 hypotheses. Through the cell flow velocity experiment, we demonstrated the importance of flow velocity control for computer vision–based cellular analysis. We also illustrated how gravity could be used to drive the flow and our bottleneck microfluidic design could slow down the flow to ensure video quality. In the cell concentration analysis experiment, we showed the design of the microfluidic chip by improving the system we used in the flow velocity experiment. We further demonstrated SmartFlow could accurately count cells and estimate cell concentrations.

We describe the following contributions in this paper: (1) the design of SmartFlow, a 3D-printed, pump-free microfluidic chip for cell concentration analysis on smartphones; (2) a comparison of 2 versions of microfluidic chips and an evaluation of their feasibility for speed control using smartphone-captured video streams; (3) experiments to evaluate the performance of SmartFlow for cell counting and concentration analysis.

### Comparison With Prior Work

In this section, we discuss current low-cost microfluidic systems, pumping systems for microfluidic chips, and computer vision algorithms for cellular analysis. Moreover, we highlight the contributions of this paper by comparing them with existing work.

#### Low-Cost Microfluidic Systems

Microfluidic systems are used to handle small amounts of liquid samples and are widely used in particle manipulation, including but not limited to particle detection and particle trapping [12,13]. Amiri et al [14] and Cha et al [15] proposed using microfluidic chips with a curved design for particle separation. Hasan et al [16] developed a polydimethylsiloxane microfluidic system to capture cancer cells. Solis-Tinoco et al [17] constructed a flexible microfluidic device to investigate live cell adhesion. Wu et al [18] used acoustic, electrophoretic, and hydrodynamic forces to prototype a microfluidic platform for cell separation. Beyond cellular manipulation, microfluidic platforms have demonstrated utility in the detection of H1N1 [19], H7N9 [20], and SARS-CoV-2 [21] viruses.

Combined with smartphones, microfluidics can enhance point-of-care diagnostic technologies. Chung et al [22] proposed a smartphone-based fluorescence microfluidic platform to detect norovirus in water samples, and Somvanshi et al [23] introduced a microfluidic paper-based aptasensor device, coupled with smartphone algorithms, to enable multiplexed detection of pathogenic bacteria. Most smartphone-based microfluidic platforms are limited to simple carriers containing biosamples due to the inaccessibility of droplet generators and syringe pumps [10]. Moreover, the use of 3D-printed microfluidics in SmartFlow offers advantages over traditional polydimethylsiloxane-based systems, such as greater flexibility, lower fabrication costs, and easier accessibility, as demonstrated by Au et al [24]. In comparison with existing smartphone-integrated systems, SmartFlow proposes using a 3D-printed, pump-free design that relies solely on gravity for fluid manipulation to eliminate the need for external components.

#### Microfluidic Pumping System

Our paper explores the methods to control microfluidic flow velocity for computer vision–based cellular analysis. Traditional flow speed control is achieved by syringe pumps, which are expensive and not ubiquitous in labs [25,26]. Therefore, previous work explored different natural power sources to drive flow in microfluidic systems [27]. Khor et al [28] and Xing et al [29] used liquid surface tension to control the flow speed. However, surface tension–driven techniques are hard to control and limited to liquid viscosity.

Prior work also focused on gravity-driven microfluidic systems. Goral et al [30] and Marimuthu and Kim [31] developed a communicating vessel–based gravity-driven system for cell cultures. Goral et al [30] used cellulose membranes to filter out unnecessary cells. Reis et al [32] used a glass siphon and multistep bioassays to quantify quantitative immunoassays. Kao et al [33] used communicating vessels to generate droplets for microfluidic analysis. Shin et al [34] designed a pressure-driven microfluidic system for colorimetric bioassays, and Gao et al [35] designed an external overflow unit to control the flow speed in a communicating vessel setting. Wang et al [36] achieved consistent flow speed using 2 siphons to keep the liquid surfaces. Limjanthong et al [37] tilted a table to power the flow in a microfluidic system for cultures of human-induced pluripotent stem cells.

Unlike gravity-driven cell culture systems and bioassays, computer vision–based microfluidic systems need to be observed under microscopes. Therefore, computer vision–based systems have stricter requirements for the flow velocity to ensure video quality, as we discussed previously. We first demonstrated that a high cell velocity could introduce motion blurriness to videos and lead to poor video quality. We then explored the feasibility of controlling the flow speed by using gravity to drive the flow and introducing a bottleneck design in the microfluidic channel. This technique can remove the high-speed cameras or syringe pumps used in previous microfluidic systems. We believe this is an important step to make smartphone-based microfluidic platforms more accessible.

#### Cellular Analysis With Computer Vision

Prior work applied computer vision algorithms to cellular analysis under microscopes. Red blood cell counting experiments can be automatically conducted using Hough transformation [38], and Lu et al [39] used cell signals in fluorescence assays to count cells in microfluidic droplets. Zeng et al [40] conducted a stained somatic cell counting experiment with traditional computer vision on dairy samples. Dima et al [41] and Shen et al [42] segmented florescence identities by setting a threshold on microscopy images.

As the prevalence of deep learning has surged in recent years, numerous studies have deployed deep learning models to analyze microfluidic cells. You Only Look Once (YOLO) [43] is a deep learning architecture proposed by Redmon et al [43] for object detection. Li et al [44] used YOLO on light scattering images to classify live and dead colonic adenocarcinoma cells. YOLO was also applied by Gardner et al [45] on inflorescence images to count cells in droplets. Moreover, Arjun et al [46] proved YOLO can be used to detect the mixing status of microfluidic droplets. High-speed cameras and convolutional neural networks can also be used in cell segmentation [47]. Lee et al [48] developed a user-friendly, fast deep learning model on cell sorting tasks in microfluidic systems. Furthermore, a convolutional neural network can be integrated into microscopic cell counting regression [49,50] and red blood cell morphology systems [51,52].

We applied dense optical flow algorithms in our study, given the ability of cells to flow through videos captured on our custom pump-free microfluidic platform. Compared with deep learning models, a dense optical flow algorithm had better interpretability, and it did not require a labor-intensive labelling process for cell segmentation. Additionally, we validated the effectiveness of our algorithms by manually counting the cells and estimating cell concentrations across samples of varying concentrations, which is considered the clinical standard for body fluid analysis.

## Methods

### Overview

SmartFlow consists of a microfluidic chip, smartphone, and microscope. One challenge with the pump-free microfluidic chip is controlling the flow velocity. If the velocity is too high, it can cause motion blur and poor video quality. Conversely, if the velocity is too low, the experiment duration can be prolonged, especially at low cell concentrations, where not every frame contains cells. To address these issues, we first conducted flow velocity experiments to answer RQ1 and RQ2 using 2 different microfluidic chip designs. Based on the findings, we designed a new microfluidic chip for the cell concentration analysis experiment, which simplified the experimental setup and answered RQ3. In this section, we discuss the smartphone app, system setup, microfluidic chip design, and process for both experiments. We also show how the 2 experiments can answer the 3 RQs.

### Fabrication

The microfluidic chips were fabricated with a PolyJet 3D printer (Stratasys), which offers high-resolution printing using photopolymers. During the printing process, the print head with nozzles applied small droplets of material to the printing plate, and ultraviolet light was used to harden the material immediately. In this way, the microfluidic chips were manufactured layer by layer. To ensure compatibility with microscopes for cellular analysis, we selected a transparent material during prototyping. This material has a light transmittance of 85% to 92% and a yellow index of 0.6 to 1.2. Additionally, water-soluble wax was used to support the hollow structures during printing. Afterward, the printed chips were immersed in water to dissolve the wax.

### Smartphone App

**Figure 1:**
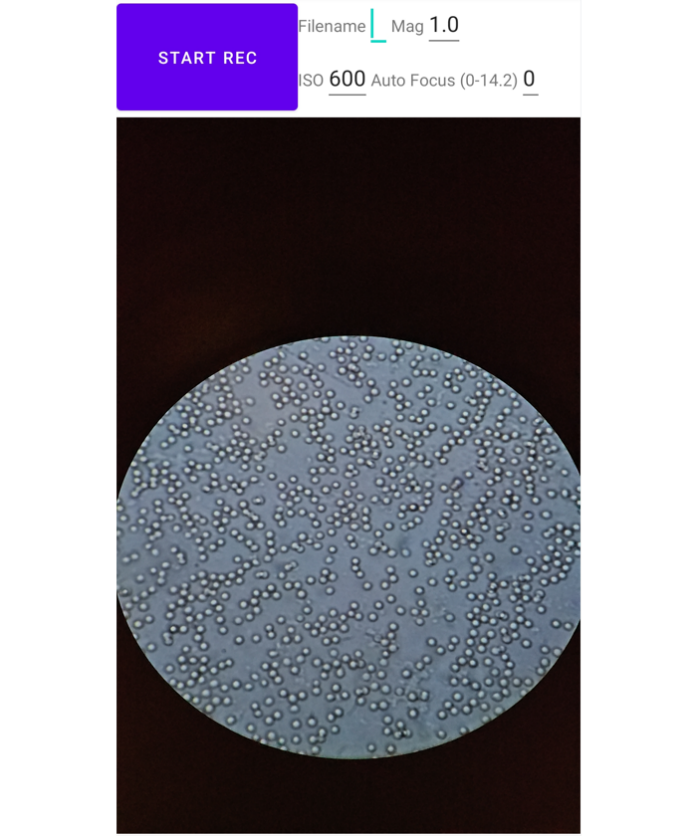
Screenshot of the smartphone app for data collection.

The smartphone app was used to record videos through the microscope’s eyepiece and was developed with Android Studio in Java. A screenshot of the developed smartphone app is illustrated in Figure 1. The camera was controlled using the Camera2 application programming interface, while the video was recorded using the MediaRecorder library.

The application operated at a frame rate of 60 frames per second (FPS) with 2K resolution (1920×1080) while visualizing the video stream. It supported inputs like ISO, zoom ratio, recordings’ file names for labelling, and focal distance, which were the key parameters to ensure video quality. We found it was important to maintain a fixed perspective without any digital zoom-in or zoom-out and set the focal distance to infinity so that any potential interference from the camera’s lens groups can be eliminated. Experimenters can record the video by pressing the start button and stop recording by pressing the button again.

### Ethics Approval

Since sheep blood was purchased (DSB250, HemoStat) for the experiment, there were no human participants nor specimens involved. Therefore, institutional review board approval was not required. Data were collected between November 2023 and June 2024.

### Cell Flow Velocity Experiment

#### Microfluidic Chip Design

**Figure 2:**
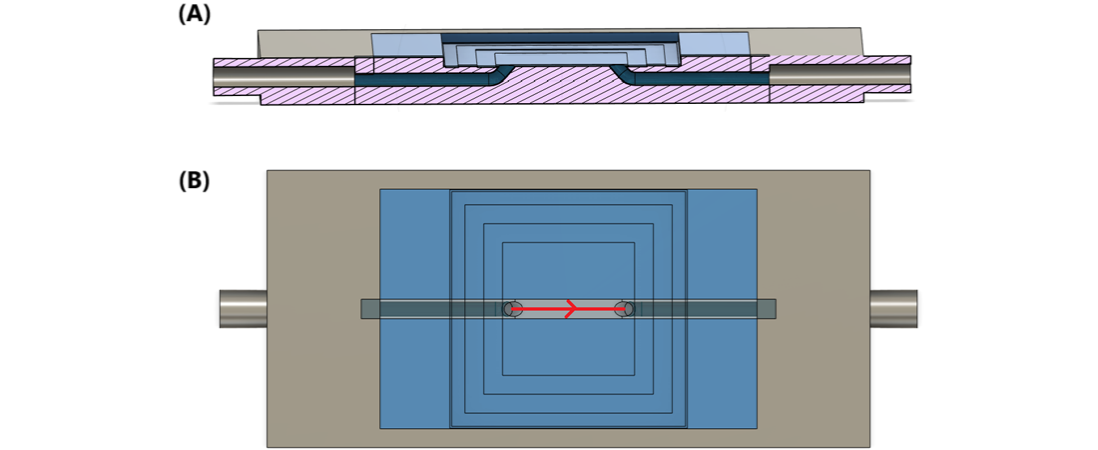
(A) Cross-sectional and (B) top views of the design for the straight microfluidic chip.

**Figure 3:**
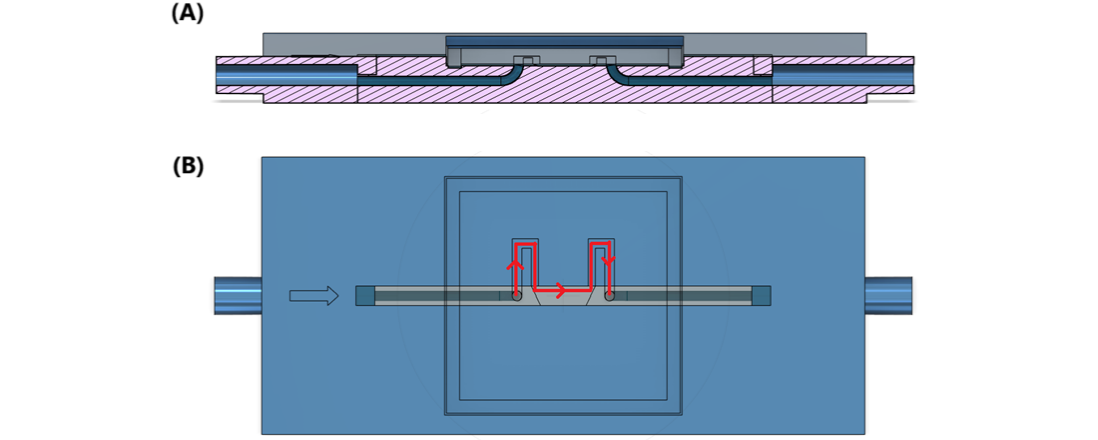
(A) Cross-sectional and (B) top views of the microfluidic design with a bottleneck.

We designed and fabricated 2 versions of microfluidic chips for this experiment, as illustrated in Figure 2 and Figure 3. Flow directions are highlighted with red arrows in the figures. Figure 2 shows the design of the straight version (version 1) of the microfluidic chip, where inlet and outlet were connected by a straight channel. The width of the channel was 2 mm, and the depth of the channel was 25 µm. However, we observed that the flow speed was highly sensitive to differences in the sample surface height, making it still challenging to control the flow velocity and maintain the video quality by adjusting the height difference.

To address this issue, we further designed a bottleneck version (version 2) of the microfluidic chip, shown in Figure 3. Introducing a bottleneck serves to slow down the flow velocity in 2 ways: First, according to Poiseuille's law, a narrower and longer channel introduces more resistance to the flow; second, the flow velocity decreases inversely with the cross-sectional area when the flow volume remains constant.

In this design, the channel remained at a depth of 25 µm. The central portion of the channel retained the same 2-mm width as the straight channel design to maintain comparability between the 2 versions. The bottleneck portion, however, was narrowed to 1 mm, reducing the cross-sectional area by one-half. This dimension was chosen to introduce sufficient resistance while still allowing cells to flow through the observation area without excessive slowing or clogging. Both microfluidic chips use slide covers sealed with ultraviolet glue to protect both the channel area and the microscope objective lens from contamination.

#### Experimental Setup

**Figure 4:**
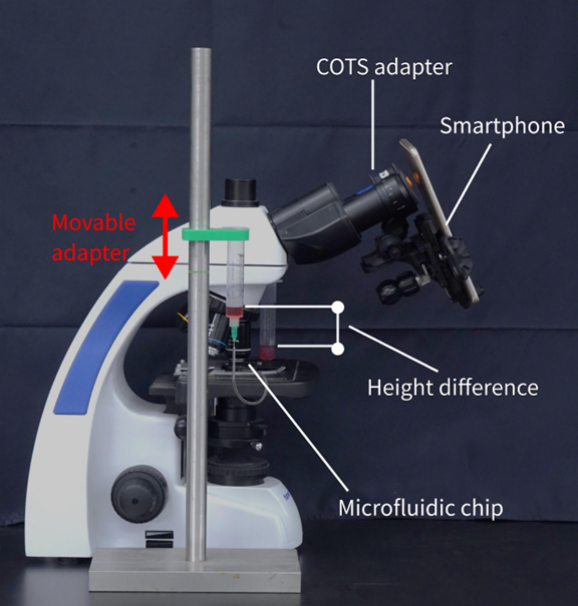
SmartFlow setup for the cell flow velocity experiment. COTS: commercial off-the-shelf.

Figure 4 illustrates the SmartFlow setup for the cell flow velocity experiment. The system was modeled on the abstraction of communicating vessels, in which gravity serves as the power source. In communicating vessels, the liquid flows to reach uniform surface heights when a height difference is created.

As shown in Figure 4, 2 syringes served as reservoirs filled with the liquid sample, and the sample flows through the microfluidic chip. In this experiment, we used 2 versions of microfluidic chips, as illustrated in Figure 2 and Figure 3. A movable syringe adapter was designed to secure the syringe on one side, and it can be moved up and down manually as illustrated by the red arrow in the figures. The purpose for this was to generate the liquid surface height difference in the 2 syringes. A 21-gauge needle was used for the syringes, with an inner diameter of 0.514 mm and an outer diameter of 0.819 mm. Needles and microfluidic chips were connected with a plastic pipe with an inner diameter of 1 mm. The needle was used on the syringe because the needle was fitted for the plastic pipe so that it could enhance airtightness at the connection junction and effectively avoid sample leakage.

The smartphone was positioned directly in front of the eyepiece of the optical microscope using a commercial off-the-shelf (COTS) adapter. The COTS adapter allowed the smartphone to move in 3 axes. We tuned the position of the smartphone to ensure the view of the microscope from the eyepiece could be captured by the smartphone. Furthermore, the microfluidic chip was put on the stage of the microscope, and we adjusted the position of the microfluidic chip using the microscope’s stage controls and focus knobs to ensure the channel on the microfluidic chip was in focus. To initiate the flow, positive pressure was applied to one side of the syringe.

#### Experimental Process

The objective of this experiment was to answer whether gravity can effectively drive the flow within the microfluidic chips, eliminating the need for external pumps or pressure systems (RQ1). In addition, we aimed to determine if the bottleneck design in the microfluidic chip reduces flow speed more effectively than straight channel designs, resulting in improved video quality for cell analysis (RQ2).

Before data collection, we captured images with gridded microscope slides (R1L3S3P, Thorlabs Inc) to establish a mapping correlation between image pixels and the actual scale observed through the microscope. We configured the microscope with an overall magnification of 1000 times, comprising a 10× magnification for the eyepiece and 100× magnification for the objective lens. The field number of the eyepieces was 22. We further calculated that the mapping correlation between the camera-captured image and the microscopy image was 5.37 pixels per µm.

We set up the entire system as shown in Figure 4 and diluted the sheep blood 10 times by mixing 0.5 mL of blood and 4.5 mL of saline. Data collection was performed using the application on the 2 chip versions, each with 7 different initial liquid surface height differences. For the straight chip, the height differences ranged from 0 cm to 0.7 cm, with increments of 0.1 cm in each trial. The height differences for the chip with the bottleneck design varied from 0 cm to 3 cm, with increments of 0.5 cm in each trial. We initially set the liquid surface to the same level, observed the flow speed at 0, and raised 1 side of the communicating vessels to the expected height difference. The cell flow velocity was estimated using a dense optical flow algorithm [53], in which dense optical flow could segment the moving objects and calculate the moving velocity and orientation of each pixel through adjacent frames in the videos.

### Cell Concentration Analysis Experiment

#### Microfluidic Chip Design

**Figure 5:**
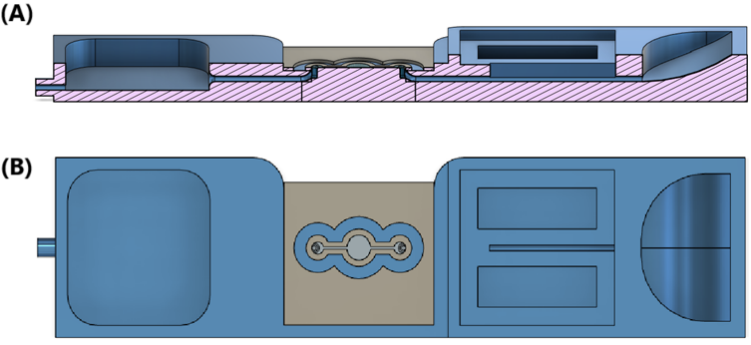
(A) Cross-sectional and (B) top views of the microfluidic chip showing, from left to right, the waste-sample reservoir, observation area, sample reservoir, and inlet. The flow direction is from right to left.

The microfluidic chip design for this experiment was based on the abstraction in the flow velocity experiment setup. Figure 5 illustrates the cross-sectional and top views of the designed microfluidic chip used in this experiment. There are 4 chambers from left to right: the waste-sample reservoir, observation area, sample reservoir, and inlet. A slide cover was used to seal the observation area to avoid contaminating the objectives. In addition, a piece of plastic film was used to seal the waste-sample chamber, which could also be used to initiate the sample flow to the observation area. When using the microfluidic chip, the sample was poured into the inlet chamber, then the sample could automatically fill the sample reservoir. The plastic film was pressed to clear the air out through the hole (leftmost hole in Figure 5), then the hole would be blocked before the plastic film was released to initiate the flow.

Flow velocity was crucial to ensure the video quality, as fast flow velocity could lead to blurriness in the videos captured by the smartphone. Therefore, we designed an overflow mechanism in the sample reservoir chamber, so the maximum liquid surface height difference between the sample reservoir and the entrance hole to the waste-sample reservoir was 2.5 mm. In addition, we learned from the flow velocity experiment that a bottleneck design could slow down the flow to ensure the cell video quality, so the same principle was applied to the observation area. The thinnest width of the channel was 0.4 mm, and the diameter of the circle in the observation area was 2 mm.

#### Experimental Setup

In the cell concentration analysis experiment, a 3D-printed microfluidic chip was placed under the microscope, while the smartphone was placed in front of an eyepiece. This setup was much simpler than the previous setup, as the syringes and plastic pipes were discarded. This was achieved by abstracting the system setup for the flow velocity experiment into the design of the microfluidic chip for the cell concentration analysis experiment. During the experiment, cell samples with different concentrations flew through the microfluidic chip, and the videos were recorded using the Android app.

#### Experimental Process

**Figure 6:**
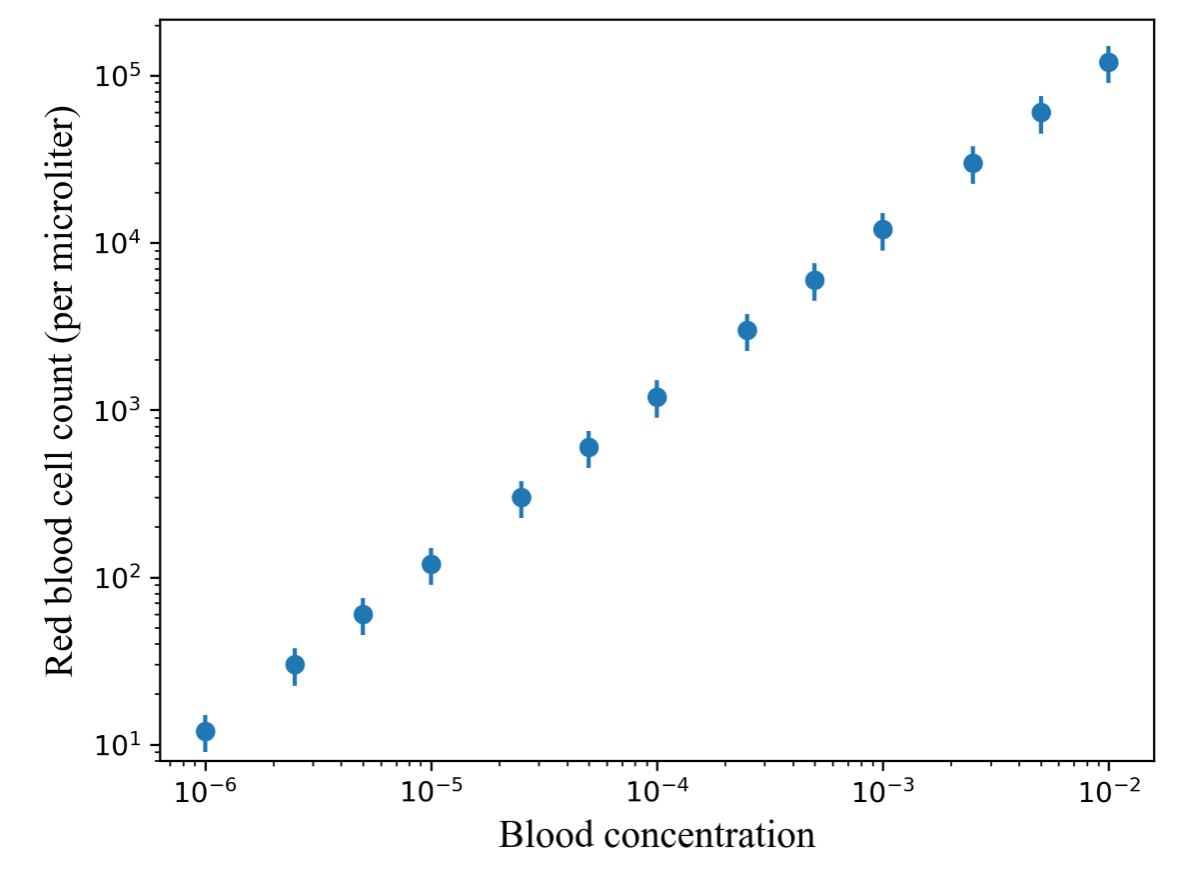
Visualization of the relationship between red blood cell count and blood concentration used for the cell concentration analysis experiment.

To conduct this experiment, we used sheep blood and diluted the blood with saline into 13 different concentrations. The red blood cell count in sheep whole blood is 9-15×10^6^ per microliter [54]. We focused exclusively on red blood cells, as the concentration of white blood cells is approximately 1000 times lower [54], making them barely observable after dilution. The concentrations used for the experiment are shown in Table 1, and the visualization is illustrated in Figure 6. The blood concentration is defined as the volume ratio between the blood and the sample. For example, a solution with a concentration of 0.1 has 1 microliter of blood contained in 10 microliters of the solution. In this way, the blood concentration before dilution is 1. In addition, the red blood cell count is defined by the number of red blood cells per microliter of the samples. These concentrations spanned a range that could be found in various body fluid tests, ranging from diluted blood samples in typical blood tests [55] to samples used for hematuria [56]. Videos were recorded via the mobile app through the eyepiece while the samples flowed through the microfluidic chip.

**Table 1:** The concentrations and red blood cell count used for the cell concentration analysis experiment.

Blood concentration	Red blood cell count (per microliter)
1×10^–^^2^	9-15×10^4^
5×10^–^^3^	4.5-7.5×10^4^
2.5×10^–^^3^	2.25-3.75×10^4^
1×10^–^^3^	9-15×10^3^
5×10^–^^4^	4.5-7.5×10^3^
2.5×10^–^^4^	2.25-3.75×10^3^
1×10^–^^4^	9-15×10^2^
5×10^–^^5^	4.5-7.5×10^2^
2.5×10^–^^5^	2.25-3.75×10^2^
1×10^–^^5^	90-150
5×10^–^^6^	45-75
2.5×10^–^^6^	22.5-37.5
1×10^–^^6^	9-15

We used 2 ground truths to explore SmartFlow’s capability to estimate cell count and concentrations in the samples (RQ3). The first ground truth involved counting the cells manually from recorded videos. The second ground truth relied on the known blood concentrations for each sample during sample preparation.

## Results

In this section, we describe the results for the flow velocity and cell concentration analysis experiments and further discuss how these experiments could answer the 3 RQs.

### Cell Flow Velocity Experiment

We explored the relationship between the height difference and flow velocity of the cells. Videos for each height difference and each type of microfluidic chip were recorded with the smartphone app, and we segmented the videos into 3 seconds of footage. The data set included 210 videos lasting 3 seconds each for the data analysis. These data were used to calculate the cell flow velocity. The dense optical flow algorithm was applied to every pair of adjacent frames within each sample. Consequently, each sample contained 3 seconds × 60 frames per second for 179 optical flow images, and the flow velocity of the cells in each sample was estimated by computing the mean of the values from the optical flow images of the cell areas. The conversion between pixels and micrometers was established through calibration using gridded microscope slides, as discussed in the Experimental Process section.

**Figure 7:**
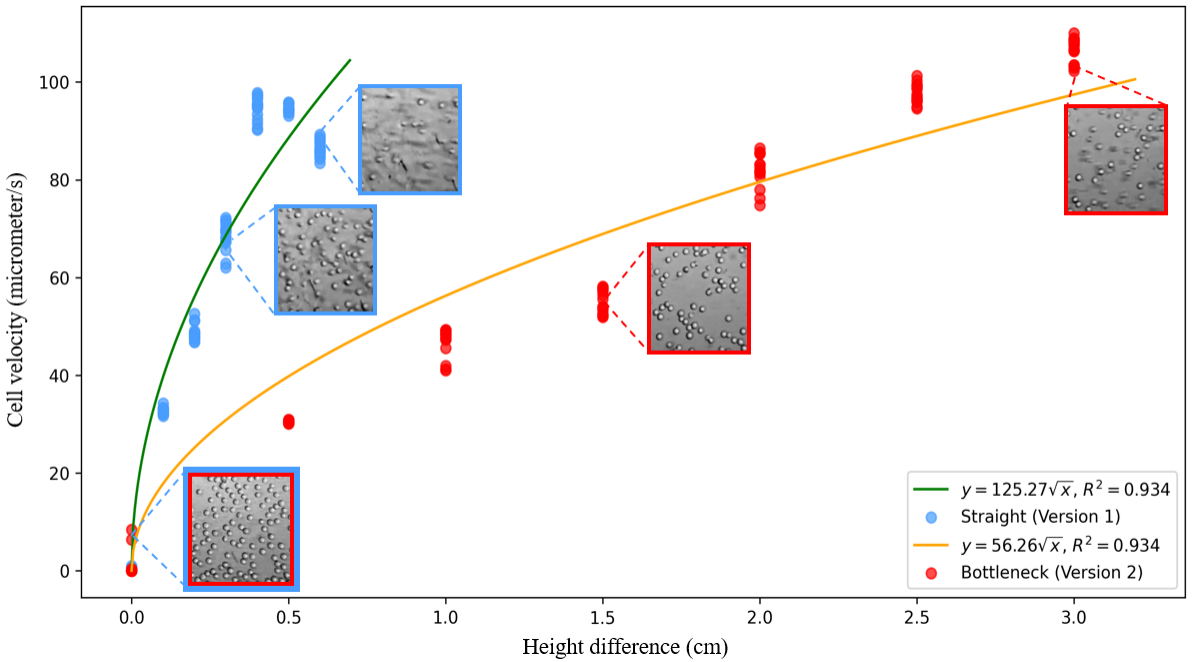
Relationship between height difference and cell velocity.

Figure 7 illustrates the relationship between the height difference and cell velocity on both microfluidic chips (version 1 and version 2). Version 1 is the microfluidic chip with the straight channel as illustrated in Figure 2, and version 2 is the microfluidic chip with the bottleneck channel as shown in Figure 3. We used the square root function to approximate the cell velocity and height difference (v=k√h, where k was the fitted coefficient). The R^2^ for both fitted curves was 0.934. Since the video footage was segmented independently, the cell velocity followed a normal distribution, and the variances were almost same, we leveraged ANOVAs to evaluate whether a height difference can influence the cell velocity. In this way, ANOVAs (version 1: *F*_6, 99_=6144.45, *P*<.001; version 2: *F*_6, 99_=3475.78, *P*<.001) showed the height difference had a significant impact on cell velocity, which answered RQ1. However, we noticed that the flow velocity decreased when height differences exceeded 4 mm on version 1 of the microfluidic chip. This was because the cell flow speed was too fast to be calculated from the videos recorded by the smartphone camera. Moreover, the images displayed in Figure 7 illustrate that motion blurriness could be introduced into the videos when the cell velocity increased.

We further performed quantitative analysis to examine the relationship between video quality and height difference. Laplacian variance (variance of the image after applying a 2D Laplacian filter) was used to determine image sharpness, where a higher variance indicates higher image sharpness. Video quality was defined as the average sharpness across all frames. To be more specific, for each video footage, Laplacian variance was applied on each gray-scale frame in the footage. The average Laplacian variance for each video footage was then calculated. Cells would not move if the height difference was 0 cm; thus, there would be no motion blurriness. To calculate the percentage video quality, we assumed the video footage with the largest averaged Laplacian variance when the height difference was 0 cm to be 100%. The video quality of the video footage was then determined by dividing the averaged Laplacian variance of the current video footage by the largest Laplacian variance when the height difference was 0. That is:




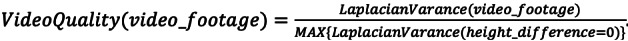




**Figure 8:**
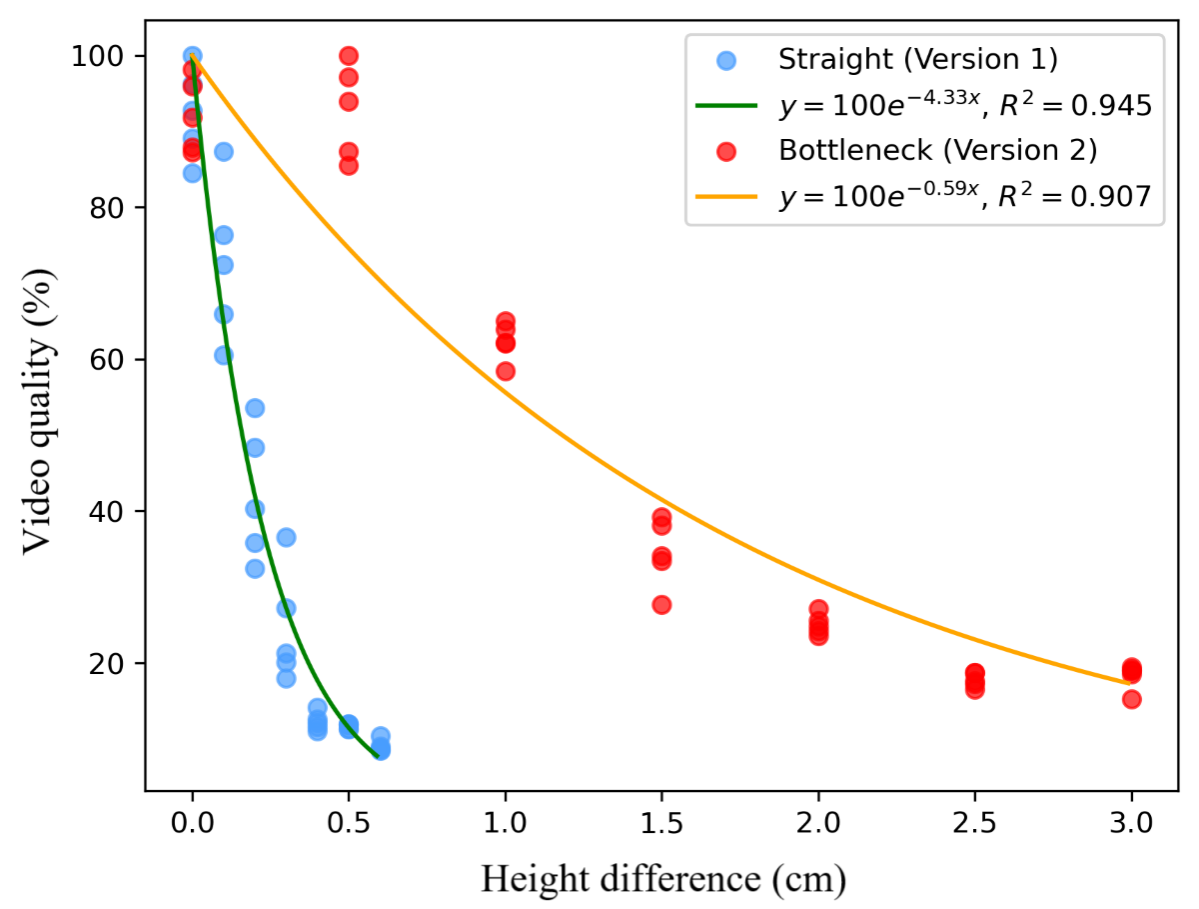
Relationship between height difference and normalized video quality.

Notice that we used the same cell concentration to conduct this experiment to avoid the potential impact introduced by different scenes. Figure 8 illustrates the relationship between height difference and video quality on both versions of the microfluidic chips, where the exponential decay functions (video quality=100*e^–k×Height^*) were used to approximate the relationship (version 1: video quality=100*e^–4.33×HeightDifference^*, R^2^=0.945; version 2: video quality=100*e^–0.59×HeightDifference^*, R^2^=0.907). Video quality was normalized by dividing by the maximum sharpness observed among all samples. Similar to the analysis on the height difference and cell velocity, videos recorded from the smartphone app were segmented into 70 videos lasting 3 seconds each. These findings answer RQ2, suggesting that bottleneck designs on microfluidic chips can decelerate flow speed, thereby enhancing video quality.

### Cell Concentration Analysis Experiment

We derived the coefficients to measure the number of cells that flowed through and cell concentrations from the video footage. The coefficient we used to measure the cells count was:




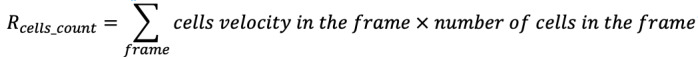




and the coefficient we used for the cell concentration prediction was:




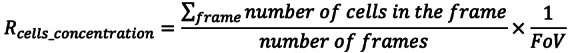




The assumption we made for the calculation was that the cells were evenly distributed in the sample.

**Figure 9:**
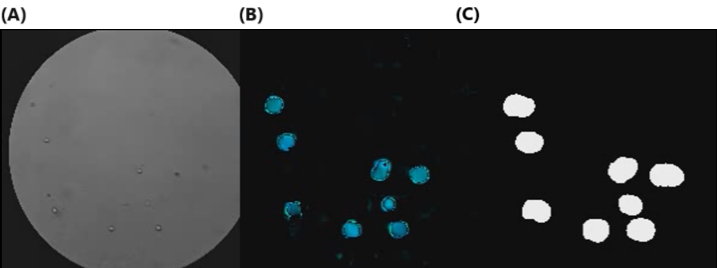
Dense optical flow visualization, including the (A) original image, (B) dense optical flow, and (C) cell segmentation.

**Figure 10:**
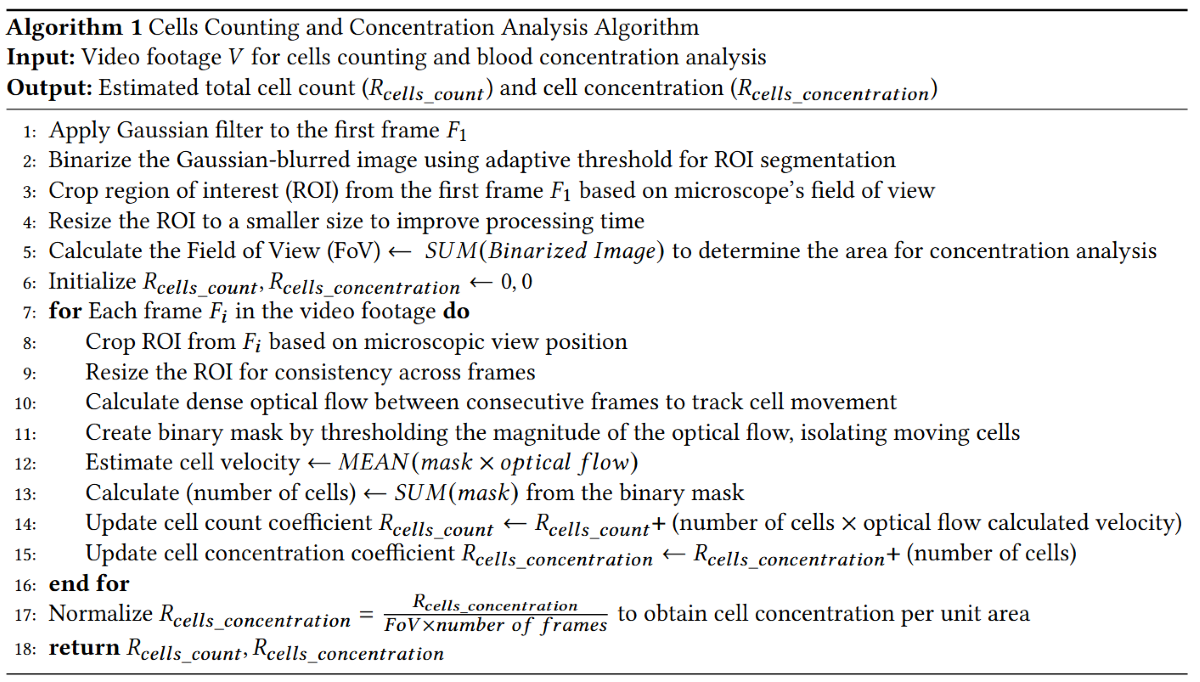
Pseudoalgorithm to calculate the cell concentration and cell count coefficients.

For each video footage, we first applied an adaptive threshold algorithm to the first frame of the video to segment the microscopic area from the entire image, and the center of the microscopic area was calculated. The number of pixels of the microscopic area were used as the field of view in the *R_cells_concentration_* calculation. The squared regions that were then cropped from the center of microscopic areas were resized to 200×200 pixels, followed by dense optical flow [53] being applied to calculate flow velocity and the number of cells in the frame. Figure 9 is a visualization of the performance of the algorithm. Similar to the flow velocity experiment, the cell velocity of each frame was calculated by taking the average of the optical flow images of the cell areas (masked by the segmented cell area). The cell velocity of each frame was estimated as the average velocity. We estimated the number of cells in each frame by calculating the number of cell pixels (white area in Figure 9C) segmented by the dense optical flow algorithm. Figure 10 shows the details of the algorithm.

**Figure 11:**
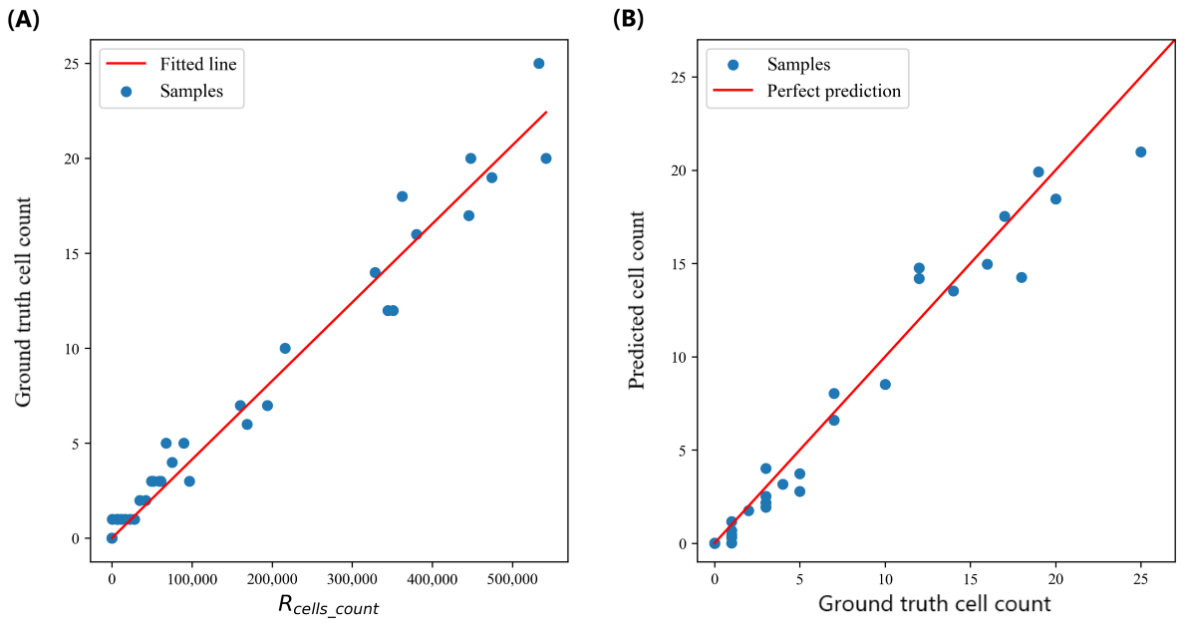
Relationships between the (A) ground truth cell count and Rcells_count and (B) predicted cell count and ground truth cell count.

To validate the cell counting performance, we manually counted the number of cells of 35 videos lasting 1 minute each, then we calculated *R_cells_count_* for each video. We used linear regression analysis to predict the number of cells in the video footage based on *R_cells_count_* (*f*(*x*)=*kx*, where *k*=4.14×10^–5^), which rendered good results as illustrated in Figure 11A (R^2^=0.97, mean absolute error=0.95, mean squared error=1.61, root mean squared error=1.27). We further used 5-fold cross-validation (28 data points for training and 7 data points for validation) to further evaluate the correlation between predicted cell count and the ground truths, as shown in Figure 11B (R^2^=0.96, mean absolute error=1.10, mean squared error=2.24, root mean squared error=1.50).

**Figure 12:**
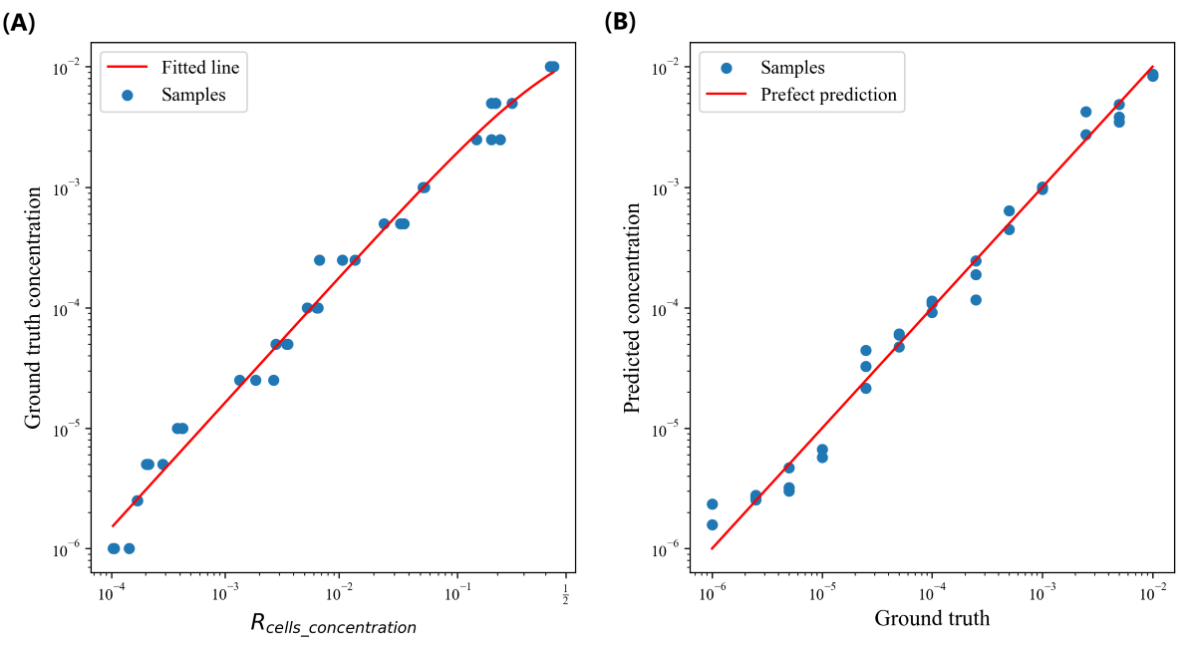
Relationships between the (A) ground truth concentration and Rcells_concentration and (B) predicted cell concentration and ground truth.

We also investigated the cell concentration regression performance of SmartFlow. We used a sliding window to first segment each recorded video. We set a window size of 150 seconds, and we used a step size of 75 seconds. We than had a total of 39 videos lasting 150 seconds each, and *R_cells_concentration_* was calculated for each video footage. To fit the curve between *R_cells_concentration_* and the ground truth concentrations, we first took the logarithm on both *R_cells_concentration_* and the ground truth concentrations and fitted the logarithms with a linear regression model (*f*(*x*)=*kx*+*b*, where *k*=1.04 and *b*=–3.85). We chose to use linear regression analysis on the logarithmic scale because the concentration differences ranged from 10^–2^ to 10^–6^, which could bias the regression model if we used a mean squared error loss function on the original scale. Figure 12A shows the fitted curve achieved an R^2^ of 0.99, mean absolute error of 0.24, mean squared error of 0.10, and root mean squared error of 0.32 on the logarithmic scale and a mean average percentage error of 0.25. We further used 5-fold cross-validation (32 data points for training and 7 data points for validating) to examine the correlation between predicted and ground truth cell concentrations, and the results are shown in Figure 12B. Our approach achieved an R^2^ of 0.99, mean absolute error of 0.24, mean squared error of 0.11, and root mean squared error of 0.33 on the logarithmic scale and a mean average percentage error of 0.25. This experiment validated SmartFlow’s capability to effectively estimate cell counts and concentrations (RQ3).

## Discussion

### Principle Findings

In this paper, we presented the design and evaluation of SmartFlow, a pump-free microfluidic platform for smartphone-based cell concentration analysis. Our system is more affordable than commercial flow cytometers and easier to set up than previous microfluidic chips, as it eliminates the need for syringe pumps and high-speed cameras by using gravity to drive the flow. We also designed microfluidic chips and conducted experiments to demonstrate the importance of controlling cell flow velocity for maintaining video quality. Additionally, an optical flow–based computer vision algorithm accurately estimated cell counts and concentrations in samples. We believe SmartFlow represents an important step toward making smartphone-based microfluidic platforms more accessible.

### Strengths and Limitations

#### Cost and Accessibility

Flow cytometers are used for cellular analysis in laboratory settings due to their high accuracy and precision. However, the high cost of these devices—ranging from US $100,000 to US $500,000—limits their accessibility, particularly in low-resource regions. Furthermore, operating a flow cytometer requires trained personnel, which further restricts their use to specialized laboratory environments. In contrast, the SmartFlow system is designed to be a low-cost, accessible alternative. The material cost for the 3D-printed microfluidic chip is approximately US $15, making it significantly more affordable. Additionally, our system is built using a COTS smartphone adapter, smartphone, and microscope, all of which are more commonly available in clinical and point-of-care settings.

#### Gravity-Driven Design

The use of gravity-driven flow in our design offers practical advantages, particularly in low-resource or point-of-care settings. By eliminating the need for external pumps or complex fluid control systems, the design simplifies the overall setup and reduces the cost and technical complexity. This makes the system more accessible for use in clinical settings, where portability and ease of use are critical. However, the reliance on gravity also introduces some challenges, as the flow becomes more sensitive to height differences. In our experiments, we addressed this by introducing a bottleneck channel design to maintain a balance between flow speed and video quality. This design approach not only improves performance in our system but can also be applied to other microfluidic applications, such as cell cultures, to achieve better flow velocity control in similar gravity-driven setups.

#### Bottleneck Design

The flow velocity was highly sensitive to changes in height, and due to the limitations of smartphone cameras, even small increases in height differences could lead to motion blur in the captured videos. The bottleneck design helped mitigate this sensitivity by controlling cell velocity. Since the flow rate (the volume of liquid passing through the channel per unit of time) remains constant throughout the channel, it is determined by the product of the cross-sectional area and flow velocity. In our design, the observation area has a larger cross-sectional area than the bottleneck, which has a smaller cross-sectional area. As a result, the flow velocity in the observation area is lower, and it becomes less sensitive to height variations. This reduction in sensitivity effectively minimizes motion blur, allowing for clearer, more stable video capture of the cells in the observation area.

#### Accuracy

We evaluated our system’s ability to predict blood concentrations using a 5-fold cross-validation method. The system achieved a mean absolute percentage error of 0.25 in predicting blood concentrations when compared with the ground truth. This demonstrates the system’s capability to perform concentration estimation. However, the accuracy of established technologies, such as flow cytometers, can serve as the ground truth in clinical settings. Therefore, we plan to explore methods to further improve the accuracy of our system by leveraging a more accurate flow volume and cell counting measurement.

### Future Directions

#### Different Cell Types

Although SmartFlow can estimate cell concentrations, it currently only applies to single types of cells. Body fluids, such as urine and blood, contain multiple cell types, and changes in cell concentrations or morphology can be valuable indicators of various diseases. Therefore, we plan to conduct studies by further mixing different types of cells and integrating object detection and segmentation deep learning models into the system to support cell classification and segmentation.

#### Liquid Flow Measurement

The concentration ground truths we used were derived from sample preparation. Even if our system can estimate the flow velocity and the number of cells passing through the video, the cell velocity might differ from the liquid’s flow velocity. Moreover, the bubbles in the microfluidic channels could potentially impact the flow dynamics. Therefore, we cannot directly estimate cell concentration by dividing the number of observed cells by the amount of liquid that flowed through. However, clinical cell counting involves various criteria, including the number of cells per microliter and number of cells per high-power field after centrifugation under a microscope. We will conduct studies to compare our system against multiple clinical standards with more samples.

#### Cell Distribution

One assumption we made when calculating cell concentration was that the cells were evenly distributed in the fluid. However, over longer experiment durations, cells could settle down in the microfluidic chip, resulting in uneven cell distribution. This effect might influence the experiment results negatively when cell concentrations are low. Therefore, we plan to collect more data on low-concentration samples and evaluate the optimal experiment duration to obtain robust results.

#### Generalizability of Body Fluids

Different biofluids can exhibit unique properties, such as viscosity, hydrophobicity, and hydrophilicity, which may interact differently with microfluidic material. Our system uses gravity to drive the flow and uses a bottleneck design to achieve better control over the flow velocity. These features are grounded in fluid dynamics principles, making them adaptable to fluids with diverse physical properties. By tuning the height differential and adjusting the bottleneck geometry, we believe the system can be optimized for a wide range of biofluids. Nonetheless, further testing is required to fully explore its applicability to fluids like saliva, urine, and cerebrospinal fluid, where more pronounced variations in viscosity or particulate matter may occur.

### Conclusions

SmartFlow is a low-cost, smartphone-based, pump-free platform that supports cell concentration regression. The bottleneck microfluidic chip can be used to effectively preserve video quality, and the proposed system could count the cells and estimate cell concentrations in the samples. We believe SmartFlow is an important stepping stone to achieving the goal of building low-cost flow cytometers for clinicians and patients by leveraging computer vision algorithms and pump-free microfluidic platforms. Beyond illustrating the feasibility of simplifying the cell concentration analysis, we envision its potential to catalyze broader innovations in the field of diagnostic technologies and contribute to the ongoing progress in body fluid analysis.

## Data Availability

The data sets generated during this study are available from the corresponding author on reasonable request.

## Abbreviations

COTS: commercial off-the-shelf
RQ: research question
YOLO: You Only Look Once
